# Glutamine Deprivation Promotes the Generation and Mobilization of MDSCs by Enhancing Expression of G-CSF and GM-CSF

**DOI:** 10.3389/fimmu.2020.616367

**Published:** 2021-02-02

**Authors:** Hong-Wei Sun, Wen-Chao Wu, Hai-Tian Chen, Yi-Tuo Xu, Yan-Yan Yang, Jing Chen, Xing-Juan Yu, Zilian Wang, Ze-Yu Shuang, Limin Zheng

**Affiliations:** ^1^ Sun Yat-sen University Cancer Center, State Key Laboratory of Oncology in South China, Collaborative Innovation Center for Cancer Medicine, Guangzhou, China; ^2^ Ministry of Education (MOE) Key Laboratory of Gene Function and Regulation, State Key Laboratory of Biocontrol, School of Life Sciences, Sun Yat-sen University, Guangzhou, China; ^3^ Department of Obstetrics and Gynecology, The First Affiliated Hospital, Sun Yat-sen University, Guangzhou, China; ^4^ Department of Breast Oncology, Sun Yat-sen University Cancer Center, Guangzhou, China

**Keywords:** MDSC, G-CSF, GM-CSF, glutamine, bone marrow

## Abstract

Solid tumors are often challenged by hypoxic and nutrient-deprived tumor microenvironments (TME) as tumors progress, due to limited perfusion and rapid nutrient consumption. While cancer cells can demonstrate the ability to survive in nutrient-deprived conditions through multiple intrinsic alterations, it is poorly understood how nutrient-deprived cancer cells co-opt the TME to promote cancer cell survival and tumor progression. In the present study, we found that glutamine deprivation markedly potentiated the expression of G-CSF and GM-CSF in mouse mammary cancer cells. The IRE1α-JNK pathway, which is activated by glutamine starvation, was found to be important for the upregulation of these cytokines. G-CSF and GM-CSF are well-known facilitators of myelopoiesis and mobilization of hematopoietic progenitor cells (HPC). Consistently, as tumors progressed, we found that several myeloid HPC compartments were gradually decreased in the bone marrow but were significantly increased in the spleen. Mechanistically, the HPC-maintaining capacity of the bone marrow was significantly impaired in tumor-bearing mice, with lower expression of HPC maintaining genes (i.e., CXCL12, SCF, ANGPT1, and VCAM1), and reduced levels of mesenchymal stem cells and CXCL12-producing cells. Furthermore, the mobilized HPCs that displayed the capacity for myelopoiesis were also found to accumulate in tumor tissue. Tumor-infiltrating HPCs were highly proliferative and served as important sources of immunosuppressive myeloid-derived suppressor cells (MDSCs) in the TME. Our work has identified an important role for glutamine starvation in regulating the expression of G-CSF and GM-CSF, and in facilitating the generation of immunosuppressive MDSCs in breast cancer.

## Introduction

Highly proliferative cancer cells exhibit a strong demand for nutrients to maintain energy supplies and for biosynthesis (1–3). However, solid tumors are often challenged by hypoxic and nutrient-deprived conditions in the tumor microenvironment (TME) due to inadequate vascular perfusion and rapid nutrient consumption as the tumor grows (4–7). When challenged with metabolic stress, cancer cells can still survive in a nutrient-poor TME through the action of multiple cancer cell-intrinsic alterations (8–10). For example, cancer cells can adapt to nutrient starvation conditions by utilizing alternative nutrients or through epigenetic modification (7, 10). In addition to cancer cell evolution, components of the TME are influential accomplices of tumor survival and progression. Therefore, it is critical to explore the impact of nutrient starvation on remodeling the TME, to better understand how the TME contributes to tumor progression.

Glucose and glutamine are fundamental nutrients used by cancer cells to meet their bioenergetic, biosynthetic, and redox demands (11–13). Although much is known about the role of aerobic glycolysis in cancer, less is understood about the role of glutamine metabolism. Glutamine is required to maintain the pool of the TCA cycle intermediate α-ketoglutarate, to support nucleoside and lipid biosynthesis, and to sustain protein glycosylation (14–17). Oncogenes, such as c-MYC and KRAS, greatly increase the uptake and catabolism of glutamine in cancer cells (18, 19), which leads to glutamine depletion in cancerous tissues (20, 21). Furthermore, nutrient competition can facilitate immune evasion of tumor cells (6, 22). However, the roles of glutamine deprivation on modulating the TME, and the underlying mechanisms that are involved, are not fully understood.

Myeloid-derived suppressor cells (MDSC) are one of the predominant components in the TME, and are known to suppress antitumor T cell responses, to support angiogenesis and metastasis, and to promote resistance to therapy (23–27). MDSCs are expanded through aberrant myelopoiesis in the bone marrow (BM), followed by mobilization and recruitment by tumor tissues (23). Tumor-produced cytokines, such as granulocyte colony-stimulating factor (G-CSF) and granulocyte/macrophage colony-stimulating factor (GM-CSF), are generally thought to induce myeloid progenitor differentiation and MDSC expansion (28–31). Thus, previous studies have aimed to decipher the regulation of these cytokines to block the generation of MDSCs (32). However, it remains unclear whether extrinsic stress, especially nutrient starvation, can stimulate cytokine production and promote MDSC expansion.

MDSC are mobilized to peripheral blood, spleen, and tumor tissues as important immune suppressors (23, 33). Moreover, our previous studies have revealed that myeloid progenitor cells are significantly increased in the peripheral blood of cancer patients, and accumulate in peripheral tissues to serve as an important source of functional MDSCs (28, 34, 35). These observations suggest that tumors systemically regulate hematopoiesis, including myeloid-biased differentiation and the mobilization of hematopoietic progenitor cells (HPCs) and immature myeloid cells. In physiological conditions, normal myelopoiesis takes place in the bone marrow and is tightly controlled by the bone marrow niche (36, 37). Although many studies have revealed the regulation of MDSCs in tumor and lymphoid tissues, the mechanism of alteration of the bone marrow niche that leads to the mobilization of immature myeloid cells in cancer remains unknown.

In the present study, we found that glutamine deprivation markedly potentiated the expression of G-CSF and GM-CSF through activating the IRE1α-JNK pathway in mouse mammary cancer cells. These cytokines were able to mobilize hematopoietic precursor cells from the bone marrow to populate peripheral tissues, especially tumor tissue. HPCs that are recruited to tumor tissues were highly proliferative and were important sources of immunosuppressive and proangiogenic MDSCs. Our work has identified an important role for glutamine starvation in regulating the expression of G-CSF and GM-CSF, and in facilitating the generation of MDSCs in breast cancer.

## Materials and Methods

### Materials

The detail of materials used in this study was summarized in **Supplementary Material, Table S1**.

### Cell Culture

4T1cells (ATCC, CRL-2539) were cultured with complete RPMI 1640 medium supplemented with 10% fetal bovine serum (FBS), penicillin (100 U/ml), streptomycin (100 mg/ml). The cells were cultured at 37°C in 5% CO_2_-humidified atmosphere.

4T1 cells were plated overnight in complete RPMI 1640 medium. For glutamine deprivation, cells were briefly washed with phosphate-buffered saline (PBS) and cultured with glutamine-free RPMI 1640 medium supplemented with or without 2 mM glutamine at the presence of 10% FBS for 24 h. For chemical treatment, cells were washed and cultured with appropriate medium supplemented with vehicle or indicated chemicals at the presence of 10% FBS for 24 h. Chemicals were used at following concentration: DON (50 μM), BPTES (10 μM), AOA (1 mM), Glucosamine (2 mM), nucleosides (1×), APY29 (10 μM), 4μ8C (2 μM), anisomycin (2.5 μg/ml), SP600125 (10 μM).

### Mouse Model

Female BALB/c mice (6–8 weeks of age) were purchased from Guangdong Medical Laboratory Animal Center (Guangzhou, China). 1×10^5^ 4T1 cells were injected subcutaneously into the flank of BALB/c mice, and tumor was grown for up to 4 weeks.

All animal experiments were performed according to state guidelines and approved by the ethical board of Sun Yat-sen University Cancer Center. All mice were maintained in the animal facilities of Sun Yat-sen University Cancer Center (Guangzhou, China) under specific pathogen-free conditions.

### Isolation of Bone Marrow Cells and Splenocytes

Bone marrow cells were harvested by flushing the femurs and tibias of each animal in PBS supplemented with 1% FBS using a 21-gauge needle. Splenocytes were obtained by homogenizing the spleen using nylon mesh. Single-cell suspension was obtained by gently aspirating several times through a 21-gauge needle. Red blood cells were removed by ACK lysis buffer. Isolated cells were then washed and resuspended for cell culture, RNA isolation or FACS analysis.

### Isolation of Tumor Infiltrating Mononuclear Cells

Tissue infiltrating mononuclear cells were obtained from fresh tumor and non-tumor tissues as described in our previous studies (34, 38). In brief, mouse tumors were cut into small pieces and digested with 0.05% collagenase type IV, 0.002% DNase I in RPMI 1640 supplemented with 20% FBS. The dissociated cells were then filtered through a 150 μm mesh and Ficoll density gradient centrifugated to obtain mononuclear cells. Isolated cells were washed and resuspended for FACS analysis or cell culture.

### Real-Time PCR Analysis

Total RNA was isolated with TRI Reagent Solution and then reverse transcribed with All-In-One RT MasterMix. Real-Time PCR was performed on LightCycler System (Roche) using SYBR qPCR Mix. Thermal cycles were: 1 min at 95°C, 40 cycles of 10 s at 95°C, 45 s at 60°C. Gene expression levels were normalized to β-Actin. The primers used are summarized in **Supplementary Material, Table S2**.

### Immunoblotting

The proteins were extracted with RIPA Lysis and Extraction Buffer and quantified with BCA Protein Assay Kit. Equal amounts of cellular proteins were separated by 10% SDS-PAGE, immunoblotted with anti p-JNK, JNK, XBP1s, CHOP, and β-Actin antibody. Antibody binding was detected using horseradish peroxidase-conjugated anti-rabbit IgG antibody and visualized with Immobilon Western Chemiluminescent kit.

### Human Subjects

Tumor tissue samples were obtained from the Sun Yat-Sen University Cancer Center. All samples were coded anonymously in accordance with local ethical guidelines (as stipulated by the Declaration of Helsinki), and written informed consent was obtained. The protocol was approved by the ethical board of Sun Yat-sen University. Fresh tumor from patients with pathologically confirmed breast cancer (n = 8) were used for immunohistochemistry staining.

### Preparation of Tissue Sections

The paraffin-embedded sections were prepared as described previously (39). In brief, tissues were formalin-fixed, paraffin-embedded, cut into 4 μm sections using a microtome, and dried. Mouse femurs and tibias were decalcified with 10% (m/v) EDTA solution (pH = 7.4) before embedment.

### Immunohistochemistry

Immunohistochemistry was performed as described previously (39). The sections were rehydrated with a decreasing ethanol series after deparaffinized with xylene. Then, the slides were soaked in 0.3% H_2_O_2_ for 10 min to quench the endogenous peroxidase activity and boiled in 10 mM citrate buffer (pH 6.0) for 10 min for heat-induced epitope retrieval. Cooled slides were washed and incubated with anti SDF1 antibody or anti G-CSF antibody overnight at 4°C. Signals were visualized with horseradish peroxidase-conjugated anti-rabbit/mouse Dako REAL™ EnVision™ detection systems (Dako, Cat# K5007) according to the manufacturer’s instructions. All sections were counterstained and mounted with a non-aqueous mounting medium. The images were captured and analyzed by optical microscope (Olympus) or Vectra-Inform image analysis system.

### Flow Cytometry

Cells cultured *in vitro*, bone marrow cells, splenocytes and tumor-infiltrating immune cells isolated from fresh samples were prepared and suspended in PBS buffer supplemented with 1% heat-inactivated FBS, then stained with desired antibodies (28, 34). For intracellular staining, the cells were stained with surface markers, fixed and permeabilized with Foxp3/Transcription Factor Staining Buffer Set, then stained with desired intracellular antibody. Data were acquired on Gallios or CytoFlex (Beckman Coulter) and analyzed with FlowJo software. The fluorochrome-conjugated antibodies used are summarized in **Supplementary Material, Table S1**.

### Hematopoietic Precursors Homing Assay

Hematopoietic progenitor homing assay was performed as described previously (40). Mice were lethally irradiated (8 Gy, one dose), One or 2 irradiated mice in each group did not receive a transplant to assess the numbers of residual host-derived progenitors. 5×10^6^ CFSE-labeled bone marrow cells from healthy BLAB/c donor mice in 200 μl PBS were intravenously injected into each mouse. After 3 h, the bone marrow cells of a femur were harvested, and a quarter of cells were transferred to colony-forming units in culture (CFU-C) assay. The number of total CFU-Cs homed to bone marrow was estimated by the number of colonies per femur (multiplied by 16.9 because one femur represents approximately 5.9% of the total murine bone marrow) (41).

### Colony-Forming Units in Culture Assay

C-Kit^+^ cells were purified from tumor-infiltrating mononuclear cells using CD117 microbeads. C-Kit^+^ cells or bone marrow cells were plated and cultured in MethoCult™ GF M3434. The types and numbers of colonies were recognized and counted according to the manufacturer’s criteria after 14–16 days of culture.

### Generation of MDSC From Hematopoietic Precursor Cells

To generate MDSCs, hematopoietic precursor cells were purified from bone marrow by CD117 microbeads kit. Purified c-Kit^+^ cells were plated at 2.5×10^5^/well in 24-well plates in complete DMEM medium (with 10% FBS) supplemented with or without 10% tumor culture supernatant, and cultured at 37°C in 5% CO_2_-humidified atmosphere for 3 days.

### Co-Culture of MDSC and Splenocytes

Mononuclear splenocytes were isolated from homogenized spleen of healthy donors by Ficoll density gradient centrifugation. These cells were stained with 2 μM CFSE for 10min at 37°C according to the manufacturer’s instructions. After CFSE staining, splenocytes were co-cultured with washed MDSCs at indicated ratio in the presence of 2.5 μg/ml coated anti-CD3 antibody, 5 μg/ml soluble anti-CD28 antibody and 20 U/ml recombinant IL-2. Cells were cultured at 37°C in a 5% CO_2_-humidified atmosphere for 3–5 days. Subsequently, the co-cultured cells were collected, stained with surface markers, and analyzed by flow cytometry.

### Statistical Analysis

IBM SPSS Software (IBM Corporation) and GraphPad Prism (GraphPad Software) were used for the statistical analysis. The significance of differences between groups was determined by the Student’s t-test or Mann–Whitney test, as appropriate. The overall survival curves were generated by the Kaplan–Meier method and analyzed using the log-rank test. *P* < 0.05 was considered significant.

## Results

### Glutamine Starvation Potentiates G-CSF and GM-CSF Expression in Breast Cancer Cells

**Figure 1:**
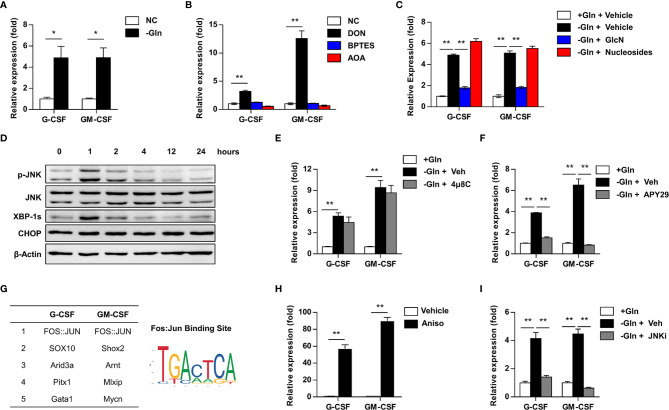
Glutamine deprivation enhances the expression of G-CSF and GM-CSF by activating IRE1α-JNK pathway. **(A)** 4T1 cells was cultured with (NC) or without (-Gln) glutamine for 24h. The expression of G-CSF and GM-CSF were determined by qRT-PCR and summarized as means ± SEM. n = 3; *P < 0.05. **(B)** The expression of G-CSF and GM-CSF were quantified in 4T1 cells treated with or without glutamine metabolism inhibitors for 24h. DON, pan inhibitor of glutamine utilization; BPTES, inhibitor of the glutaminase GLS1; AOA, inhibitor of aminotransferase. n = 5; **P < 0.01. **(C)** 4T1 cells were cultured in complete medium (+Gln +Veh) or glutamine deprived medium (-Gln) supplemented with vehicle(+Veh), glucosamine(+GlcN) or nucleosides. The expression of G-CSF and GM-CSF were shown as means ± SEM. n = 4; **P < 0.01. **(D)** Western blot showed the level of p-JNK, JNK, XBP-1s, and CHOP in 4T1 cells after glutamine starvation. **(E, F)** The expression of G-CSF and GM-CSF were quantified in glutamine deprived 4T1 cells supplemented with glutamine, or treated with vehicle, 4μ8C (inhibitor of IRE1α endoribonuclease activity) **(E)** or APY29 (inhibitor of IRE1α kinase activity) **(F)** for 24 h. n = 3 in **(E)**, n=4 in **(F)**; **P < 0.01. **(G)** Transcript factors that could bind to the promoter of G-CSF and GM-CSF were predicted by JASPAR database. **(H)** 4T1 cells were cultured in complete medium treated with vehicle or anisomysin (JNK agonist) for 24 h. The expression of G-CSF and GM-CSF were summarized as means ± SEM. n = 3; **P < 0.01. **(I)** 4T1 cells were cultured in complete medium or glutamine-deprived medium treated with vehicle or SP600125 (JNK antagonist) for 24 h. The expression of G-CSF and GM-CSF were shown as means ± SEM. n = 3; **P < 0.01.

Solid tumors are challenged by a nutrient deprived tumor microenvironment during tumor progression, due to rapid consumption and limited perfusion of nutrients (4–7). To discover the response of starved tumor cells, mouse 4T1 mammary cancer cells were cultured under glutamine-deprived conditions. The expression of G-CSF and GM-CSF was significantly upregulated in glutamine-deprived cells compared to cells cultured under normal growth conditions (**Figure 1A**). 6-diazo-5-oxo-L-norleucine (DON) is an inhibitor of several glutamine utilizing enzyme, including glutaminase, cytidine triphosphate synthase, and L-Glutamine–D-fructose-6-phosphate transaminase (12). DON treatment enhanced G-CSF and GM-CSF expression in 4T1 cells, suggesting that DON treatment phenocopies the effects of glutamine deprivation (**Figure 1B**).

Glutamine is an important metabolite involved in mitochondrial metabolism, synthesis of nucleosides, and protein glycosylation. To elucidate the metabolic processes involved in regulating the expression of G-CSF and GM-CSF, multiple selective inhibitors and metabolic products were introduced into 4T1 culture medium. Bis-2-(5-phenylacetamido-1,3,4-thiadiazol-2-yl) ethyl sulfide (BPTES) and aminooxyacetic acid (AOA) are inhibitors of the kidney-type glutaminase isoform (GLS1) and aspartate aminotransferase (42, 43), which catalyze glutaminolysis. In contrast to DON, BPTES and AOA treatment did not phenocopy the effects of glutamine deprivation, indicating that glutaminolysis is not involved in regulating the expression of G-CSF and GM-CSF (**Figure 1B**). Supplementation of glucosamine, rather than nucleoside supplementation, attenuated the expression of G-CSF and GM-CSF induced by glutamine deprivation (**Figure 1C**). Collectively, these data suggested that glutamine deprivation potentiates the expression of G-CSF and GM-CSF independent of glutaminolysis.

### JNK Activation Contributes to Enhanced G-CSF and GM-CSF Expression in Glutamine-Deprived Cells

Glutamine-derived hexosamine synthesis is important for protein glycosylation and endoplasmic reticulum homeostasis; we therefore evaluated the effects of glutamine starvation on the endoplasmic reticulum stress pathway. After glutamine deprivation, XBP1s and JNK were rapidly upregulated, while CHOP expression was not changed, indicating activation of the IRE1 pathway, rather than the PERK pathway (**Figure 1D**). IRE1 contains two functional domains, the kinase domain contributes to autophosphorylation and the activation of JNK pathway, and the endoribonuclease domain is known for splicing of XBP1 mRNA (44, 45). To identify the functional pathway involved in IRE1 regulation of G-CSF and GM-CSF expression, specific inhibitors of either IRE1 kinase activity or IRE1 endoribonuclease activity were applied to the glutamine-deprived cells. The kinase inhibitor APY29 (46) significantly blocked the upregulation of G-CSF and GM-CSF in glutamine-starved 4T1 cells, but 4μ8C (47), the endoribonuclease inhibitor, did not (**Figures 1E, F**).

Additionally, we used the JASPAR database to analyze the promoter regions of G-CSF and GM-CSF (48) and we found that a transcription factor downstream of JNK, the Fos-Jun complex, could bind to the promoters of both G-CSF and GM-CSF (**Figure 1G**). Consistently, the JNK agonist anisomycin (45) could mimic the effects of glutamine starvation in complete medium (**Figure 1H**). The JNK antagonist SP600125 (49) could completely inhibit the upregulation of G-CSF and GM-CSF in glutamine-starved 4T1 cells (**Figure 1I**). These data suggested that the IRE1-JNK pathway is activated by glutamine starvation and is critical to the upregulation of G-CSF and GM-CSF in glutamine-deprived 4T1 cells.

### Hematopoietic Precursor Cells Are Mobilized From the Bone Marrow in Tumor-Bearing Mice

**Figure 2:**
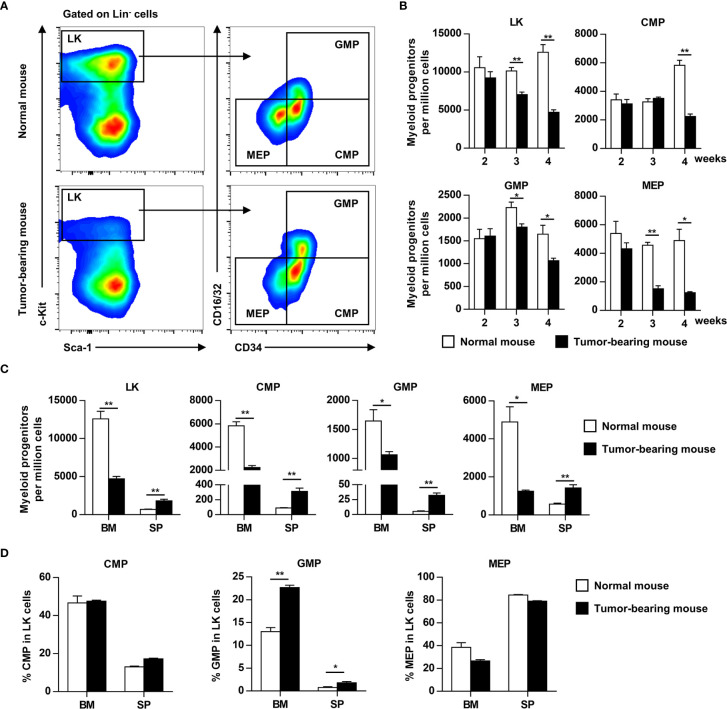
Myeloid progenitors are mobilized from bone marrow to the periphery as tumor progresses. **(A)** Myeloid progenitor subsets in the bone marrow (BM) of normal or tumor-bearing mice were analyzed by flow cytometry 4 weeks after tumor inoculation. LK, Lineage^-^c-Kit^+^ cells; CMP, common myeloid progenitors; GMP, granulocyte–macrophage progenitors; MEP, megakaryocyte–erythroid progenitors. **(B)** The number of myeloid progenitor subsets in the bone marrow of normal or tumor-bearing mouse at indicated time after tumor inoculation were shown as means ± SEM. n = 3~5/group; *P < 0.05; **P < 0.01. **(C)** The number of myeloid progenitors in bone marrow and spleen of normal or tumor-bearing mouse 4 weeks after tumor implantation were summarized as means ± SEM. n = 3; *P < 0.05; **P < 0.01. **(D)** The proportion of CMP, GMP, and MEP in LK cells of bone marrow and spleen from normal or tumor-bearing mouse were summarized as means ± SEM. n = 3; *P < 0.05; **P < 0.01.

G-CSF and GM-CSF are important cytokines involved in myelopoiesis and HPC mobilization. To determine the effects of elevated G-CSF and GM-CSF expression on hematopoiesis, the HPC compartment was investigated in the bone marrow of tumor-bearing mice. HPCs (Lin^-^Sca-1^-^c-Kit^+^ cells) were gradually decreased in the bone marrow of tumor-bearing mice, and the decrease became more pronounced as tumor progression continued (**Figures 2A, B**). The decrease of LKs in bone marrow coincided with a reduction in common myeloid progenitors (CMP), granulocyte/macrophage progenitors (GMP), and megakaryocyte/erythroid progenitors (MEP) (**Figure 2B**). By contrast, LK, CMP, GMP, and MEP were markedly increased in the spleens of tumor-bearing mice (**Figure 2C**). Changes in the distribution of HPCs suggest that growing tumors mobilized hematopoietic precursor cells from the bone marrow to the periphery. Moreover, the proportion of GMP in hematopoietic precursor cells from the bone marrow and spleen of tumor-bearing mouse was markedly increased compared to normal mouse (**Figure 2D**).

**Figure 3:**
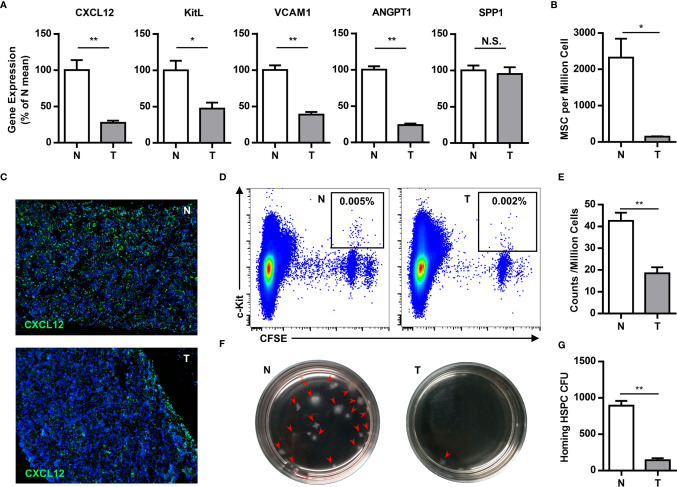
Tumor impairs the residence of hematopoietic precursor cells in the bone marrow. **(A)** The expression of genes that regulate HPCs maintenance and attraction (Cxcl12, c-kit ligand, angiopoietin-1, vascular cell adhesion molecule-1 and osteopontin) in the bone marrow of normal (N) or tumor-bearing (T) mice were quantified by qPCR and summarized as means ± SEM. n = 5; *P < 0.05; **P < 0.01. **(B)** The number of mesenchymal stem cells (CD45^-^Lin^-^CD34^-^c-kit^-^CD29^+^) in the bone marrow of normal (N) or tumor-bearing (T) mice were detected by flow cytometry and summarized as means ± SEM. n = 3; *P < 0.05. **(C)** Immune staining showed the CXCL12 expression in the bone marrow of normal (N) or tumor-bearing (T) mice. **(D)** 5×10^6^ CFSE labeled bone marrow cells were intravenously injected into lethally irradiated normal or tumor-bearing mouse. Labeled hematopoietic precursor cells homing to the bone marrow of normal (N) or tumor-bearing (T) mice after 3h were analyzed by flow cytometry. **(E)** The proportion of labeled precursors in **(D)** was summarized as means ± SEM. n = 5; **P < 0.01. **(F)** Colony forming assay showed the hematopoietic precursors homing to the bone marrow of normal (N) or tumor-bearing (T) mice. **(G)** The colony units in **(F)** were calculated and summarized as means ± SEM. n = 5; **P < 0.01.

The bone marrow is the primary site of HPC maintenance and hematopoiesis in adults. To gain more insight into the mobilization of HPCs, we analyzed the expression of genes that regulate HPC maintenance and attraction in the bone marrow (CXCL12, c-kit ligand, angiopoietin-1, vascular cell adhesion molecule-1 and osteopontin) in tumor-bearing mice. The expression of these genes, with the exception of osteopontin, was 2–4 fold downregulated in the bone marrow of tumor-bearing mice (**Figure 3A**). Meanwhile, the key components of the bone marrow niche, mesenchymal stem cells (CD45^-^Lin^-^CD34^-^c-kit^-^CD29^+^) and CXCL12 producing cells, were significantly decreased in the bone marrow of tumor-bearing mice (**Figures 3B, C**).

To evaluate the impact in progenitor trafficking in the bone marrow, we assayed hematopoietic progenitor homing to the bone marrow. Carboxyfluorescein diacetate succinimidyl ester (CFSE)-labeled bone marrow cells from congenic donor mice were intravenously injected into lethally irradiated normal or tumor-bearing mouse. Compared to normal mice, CFSE labeled c-kit^+^ precursors and hematopoietic colony-forming cells, homing to the bone marrow, were markedly reduced in tumor bearing mice (**Figures 3D–G**). These data demonstrate that breast cancer growth leads to perturbations in the bone marrow niche, resulting in mobilization of HPCs to the periphery.

### Hematopoietic Precursor Cells and Myeloid-Derived Suppressor Cells Are Highly Enriched in Mouse Mammary Tumors

**Figure 4:**
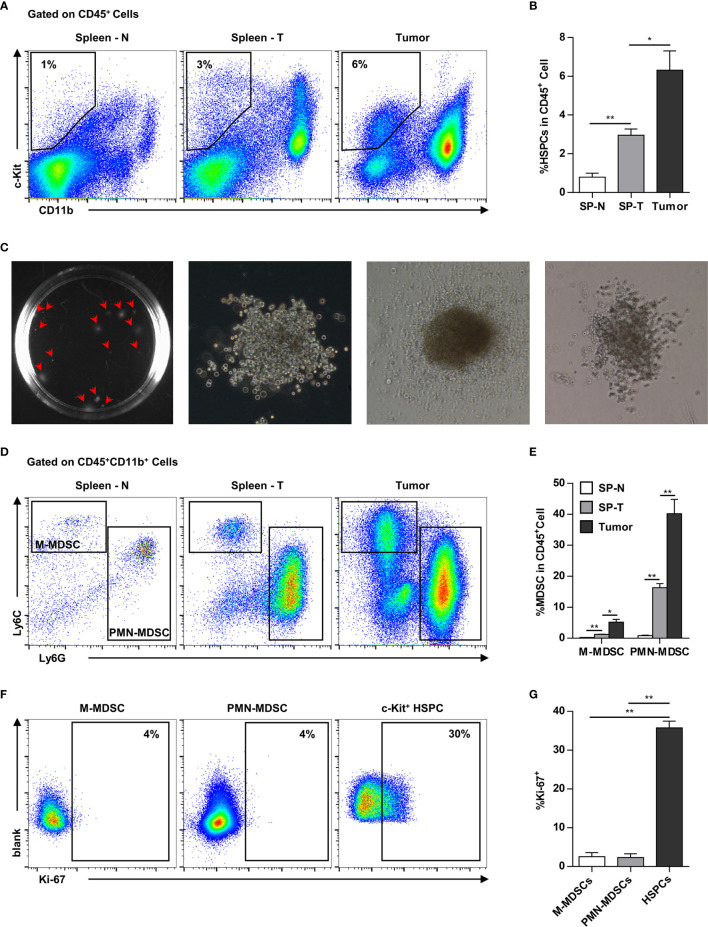
Myeloid progenitor cells are further enriched in the tumor tissues. **(A, B)** Representative flow cytometry showed the proportion of CD11b^-^c-Kit^+^ hematopoietic precursors in the spleen of normal mice (Spleen - N; SP-N), the spleen (Spleen - T; SP-T) and tumor tissue of tumor-bearing mice **(A)**. The percentage of hematopoietic precursor cells was summarized as means ± SEM in **(B)**. n = 3; *P < 0.05; **P < 0.01. **(C)** Colony forming assay showed the myelopoiesis capacity of tumor-infiltrating precursors. **(D, E)** The proportion of M-MDSC and PMN-MDSC in the spleen of normal mice (Spleen - N; SP-N), the spleen (Spleen - T; SP-T) and tumor tissue of tumor-bearing mice were analyzed by flow cytometry **(D)** and summarized as means ± SEM in **(E)**. n = 3; *P < 0.05; **P < 0.01. **(F, G)** The proliferation of M-MDSC, PMN-MDSC, and HPC in the tumor tissues were analyzed by flow cytometry **(F)** and summarized as means ± SEM in **(G)**. n = 4; **P < 0.01.

In addition to the spleen, HPCs (CD11b^-^c-kit^+^) also infiltrated into the tumor tissues. In addition to the increase of HPCs in the spleen (normal: ~1%, tumor-bearing: ~3%), HPCs were also enriched in the tumor tissues (~6%) of tumor-bearing mice (**Figures 4A, B**). The tumor-infiltrating c-kit^+^ cells were capable of myelopoiesis, as demonstrated by a colony-forming assay (**Figure 4C**). Consistently, the proportion of MDSCs, including M-MDSCs and PMN-MDSCs, was markedly increased in the spleens of tumor-bearing mice, and was even higher in tumor tissues (**Figures 4D, E**). Tumor-infiltrating MDSCs were rarely capable of proliferation, while approximately 30% HPCs were proliferating within the tumor tissue (**Figures 4F, G**). These results suggested that tumor-infiltrating HPCs are important sources of MDSCs that accumulate in the tumor tissue.

### Tumor Cells Induce HPCs to Generate MDSCs, Promoting Immune Evasion of Tumor Cells

**Figure 5:**
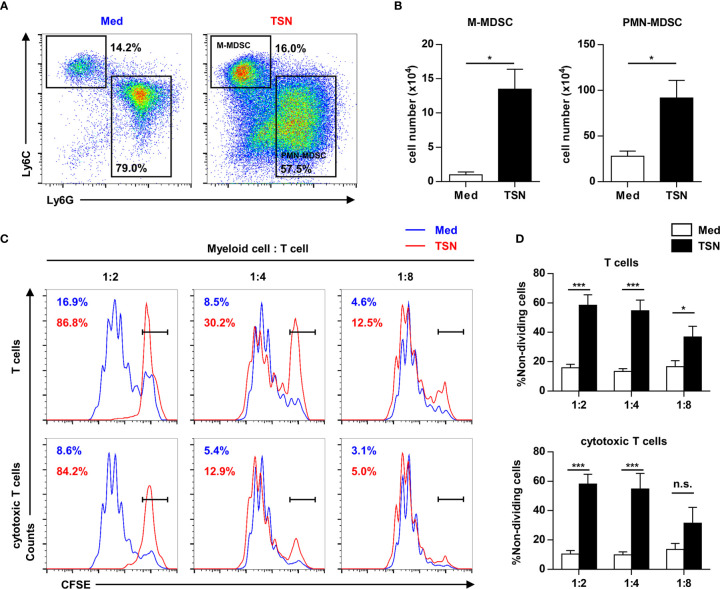
Tumor supernatant could expand tumor-infiltrating precursors into myeloid-derived suppressor cells. **(A)** 2.5×10^5^ purified c-Kit^+^ precursors were cultured with or without 10% tumor supernatant (TSN) for 3 days. The phenotype of cultured cells was analyzed by flow cytometry. **(B)** The number of monocytic (M) and polymorphonuclear (PMN) myeloid-derived suppressor cells (MDSC) in **(A)** was summarized as means ± SEM. n = 5; *P < 0.05. **(C)** Cells cultured in **(A)** were coculture with CFSE labeled splenocytes at indicated ratio in the presence of anti-CD3 and anti-CD28 antibody. The proliferation of T cells was analyzed by flow cytometry. **(D)** The proportion of non-dividing T cells and cytotoxic T cells was summarized as means ± SEM. n = 5; *P < 0.05; ***P < 0.001.

To reveal the direct influence of tumor cells on tumor-infiltrating HPC differentiation and function, purified HPCs were cultured with or without 10% tumor culture supernatant for 3 days. Most of HPCs cultured in both conditions differentiated into myeloid descendants with MDSC phenotypes (**Figure 5A**). Exposure to the tumor cell culture supernatant resulted in a significant expansion of the MDSC population (**Figure 5B**). Subsequently these myeloid descendants were co-cultured with activated splenocytes to assess their immunosuppressive capacity. MDSCs induced by tumor supernatant significantly inhibited the proliferation of T cells, especially cytotoxic T cells (**Figures 5C, D**). These data suggest that tumor-infiltrating HPCs can expand to immune-suppressive MDSCs in tumor tissue.

### High G-CSF Expression Is Significantly Associated With Poor Overall Survival in Human Breast Cancer Patients

**Figure 6:**
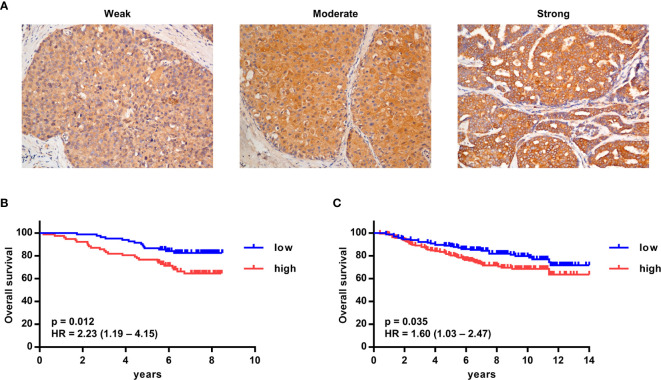
High G-CSF expression was significantly associated with decreased overall survival in human breast cancer patients. **(A)** Immunohistochemistry showed different expression levels of G-CSF in human breast cancer patients. **(B, C)** Kaplan–Meier plots of overall survival (OS) rates of breast cancer patients from two GEO data set (GSE1456 [B] and GSE20685 [C]) stratified by G-CSF expression. *P* value and hazard ratio (HR) was calculated by the log-rank test.

To detect the expression of G-CSF in tumor tissue, G-CSF was stained by immunohistochemistry in paraffin-embedded tumor sections from breast cancer patients. The expression of G-CSF was evaluated as weak, moderate, or strong (**Figure 6A**, representative staining images). In a larger set of human breast cancer samples from two publicly available datasets from the NCBI GEO database (GSE1456 and GSE20685), we tested the correlation of G-CSF mRNA expression and overall survival. High G-CSF expression was significantly correlated with poorer overall survival compared with low G-CSF expression (GSE1456, hazard ratio [HR] = 2.23, 95% confidence interval [CI] = 1.19 – 4.15, p = 0.012; GSE20685, HR = 1.60, 95% CI = 1.03 – 2.47, p = 0.035) (**Figures 6B, C**).

## Discussion

Myeloid derived suppressor cells (MDSC) are one of the predominant components in the TME, where they can suppress antitumor T cell responses (50). Better understanding of what leads to the accumulation of MDSCs in tumor tissues and the underlying mechanisms involved would inspire therapies that may abrogate an immunosuppressive TME. It has previously been unclear how glutamine-deprived conditions modulate the expansion and mobilization of MDSCs in breast cancer. In the present study, we dissected the impact of low glutamine on regulating the expression of G-CSF and GM-CSF in mouse mammary tumor cells, we elucidated the role of the bone marrow niche in the mobilization of myeloid precursors in breast cancer, and we provide evidence to mechanistically link microenvironmental glutamine depletion in cancer to the expansion and mobilization of MDSCs. Our study provides important evidence that glutamine deficiency in tumor tissues leads to expansion and mobilization of MDSCs by potentiating the expression of tumor-derived G-CSF and GM-CSF.

Solid tumors are challenged by highly hypoxic and nutrient-deprived tumor microenvironments, due to rapid nutrient consumption and limited perfusion (4–7). Hypoxia contributes to drug resistance and accumulation of immune suppressive cells (4, 51, 52). Nutrient depletion in solid tumors also has profound impacts on both cancer cells and tumor-infiltrating immune cells (7, 22, 53). Glutamine is one of the fundamental nutrients for cancer cell survival and proliferation, glutamine uptake and consumption are markedly increased in tumors. Increased glutamine catabolism depletes the local supply and leads to glutamine deprivation in the tumor tissues, including breast cancer, pancreatic cancer and sarcomas (7). Glutamine deficiency can promote dedifferentiation and drug resistance of melanoma cells through inhibition of histone demethylation (7). However, it is unclear what links tumor cell responses to glutamine deficiency to modulation of tumor microenvironment. Here, we found that glutamine deprivation markedly increased the expression of G-CSF and GM-CSF in mouse mammary cancer cells *in vitro*. It was reported that the level of G-CSF and GM-CSF were significantly upregulated in 4T1 tumor-bearing mice compared to normal mice (54). These cytokines systemically promote the expansion and mobilization of immature myeloid cells in tumor-bearing mice by impairing the maintenance capacity of hematopoietic progenitor cells in the bone marrow. After egress from the bone marrow, we found that the myeloid precursor cells were recruited to tumor tissues, where they act as immune regulators. Moreover, MDSCs could promote angiogenesis by secreting vascular endothelial growth factor and matrix metallopeptidase 9 (50). The expansion and mobilization of MDSCs is a potential response of solid tumors to improve vascular perfusion under glutamine deficient conditions. In the present study, glutamine deprivation was used to represent the nutrient-deficient condition in tumor tissues, it should be noted that other factors, such as tryptophan and arginine, may also contribute to the results.

Glutamine contributes to multiple catabolic and anabolic metabolic pathways in cancer cells, and the effects of glutamine metabolism on cell signaling, proliferation, and differentiation are becoming more clear (12, 55). We found that treatment of breast cancer cells *in vitro* with DON, an inhibitor of several glutamine utilizing enzymes, phenocopied the effects of glutamine deprivation and led to an increase in G-CSF and GM-CSF expression. These data collectively suggested that blocking glutamine metabolism in cancer cells potentiates the expression of G-CSF and GM-CSF. Glutamine is an important resource for mitochondrial metabolism, synthesis of nucleosides, and glycosylation of proteins (14–17). We therefore applied several selective inhibitors and metabolic products to determine which metabolic processes are involved in regulating the expression of G-CSF and GM-CSF. Blocking glutaminolysis process by BPTES and AOA had negligible effects on G-CSF and GM-CSF expression. On the other hand, supplementation of glucosamine in glutamine deprived medium abrogated the upregulation of G-CSF and GM-CSF. Our results revealed that the IRE1-JNK pathway is activated by insufficient hexosamine biosynthesis and is responsible for the increase of G-CSF and GM-CSF expression in glutamine-deprived mammary cancer cells. Our data link glutamine starvation to the activation of the IRE1-JNK pathway and expression of G-CSF and GM-CSF.

Myeloid derived suppressor cells (MDSC) are one of the predominant components in the tumor microenvironment, and suppress antitumor T cell responses, support angiogenesis and metastasis, and promote resistance to cancer therapy (23–27). Tumor-derived cytokines, including GM-CSF and G-CSF, are potent factors that promote myeloid progenitor differentiation and MDSC expansion (28–31). However, in the bone marrow, these cytokines promote the generation of normal myeloid cells, rather than suppressive myeloid cells (56, 57). We assumed that the niche for myelopoiesis determined the function of myeloid descendants. Our data here suggest that stromal cells and molecules involved in the maintenance and attraction of HPCs are markedly decreased in the bone marrow niche of tumor-bearing mice. Hematopoietic progenitor homing assays revealed that HPC maintenance capacity was impaired in the bone marrow of tumor-bearing mouse. Furthermore, hematopoietic precursor cells and immature myeloid cells were found to egress from the bone marrow niche of tumor-bearing mice and expanded to MDSCs in peripheral tissues. Our data demonstrate that the impaired HPC maintenance capacity contributed to extramedullary hematopoiesis and MDSC expansion in tumor-bearing hosts.

It has been reported that MDSCs are substantially enriched in late stage cancer compared to early stage cancer (58, 59). Previous studies revealed that oncogenic KRAS contributes to the production of GM-CSF and to the expansion of MDSCs in a mouse model of pancreatic ductal adenocarcinoma (32). Nevertheless, the oncogene in a cancer cell is a relatively constant factor and is not sufficient for the continuous accumulation of MDSCs. We found that the expression of G-CSF and GM-CSF is regulated by glutamine concentration in the tumor microenvironment, which is a flexible factor determined by tumor size and perfusion. Our study suggests that nutrient deficiency contributes to enhance the expression of G-CSF and GM-CSF and promotes MDSC expansion as a tumor grows.

Our study dissected the impact of low glutamine on regulating the expression of G-CSF and GM-CSF in mouse mammary tumor cells. However, our study is at preclinical stage and future investigation in human models would be required to improve the translational impact. For example, antiangiogenic therapy is known for causing limited perfusion and deficient nutrients in tumor tissues. We found that glutamine deprivation could promote the generation and mobilization of MDSCs by enhancing G-CSF and GM-CSF expression. Accordingly, previous study found that MDSCs are enriched in the tumors treated with anti-VEGFA antibody and contribute to refractoriness to antiangiogenic therapy (60). Therefore, our findings might reveal a potential pathway that mediate tumor refractoriness to anti-VEGF therapy. Considering that glutamine could also support the tumor growth, it might not be an optimal strategy to abrogate this process by glutamine supplementation. Our data suggested that the IRE1α kinase inhibitor APY29, which could block the upregulation of G-CSF and GM-CSF in glutamine deprived tumor cells *in vitro*, might be an option for future studies to reduce the mobilization and generation of MDSC.

## Data Availability Statement

The original contributions presented in the study are included in the article/**Supplementary Material**. Further inquiries can be directed to the corresponding authors.

## Ethics Statement

The studies involving human participants were reviewed and approved by the ethical board of Sun Yat-sen University. The patients/participants provided their written informed consent to participate in this study. The animal study was reviewed and approved by The ethical board of Sun Yat-sen University Cancer Center.

## Author Contributions

H-WS and W-CW designed and conducted experiments, and wrote the manuscript. Y-TX, Y-YY, and JC conducted experiments. H-TC, X-JY, ZW, and Z-YS collected human samples. LZ and Z-YS designed and supervised the research and revised the manuscript. All authors contributed to the article and approved the submitted version.

## Funding

This work was supported by project grants from the National Key R&D Program of China (2017YFA0505803 and 2018ZX10302205), the National Natural Science Foundation of China (81802663 and 81730044), and the China Postdoctoral Science Foundation (2019M653190).

## Conflict of Interest

The authors declare that the research was conducted in the absence of any commercial or financial relationships that could be construed as a potential conflict of interest.

## References

[1] DeBerardinis RJ Chandel NS Fundamentals of cancer metabolism. Sci Adv (2016) 2:e1600200. 10.1126/sciadv.1600200 27386546 PMC4928883

[2] Pavlova NN Thompson CB The emerging hallmarks of cancer metabolism. Cell Metab (2016) 23:27–47. 10.1016/j.cmet.2015.12.006 26771115 PMC4715268

[3] DeBerardinis RJ Lum JJ Hatzivassiliou G Thompson CB The biology of cancer: metabolic reprogramming fuels cell growth and proliferation. Cell Metab (2008) 7:11–20. 10.1016/j.cmet.2007.10.002 18177721

[4] Jain RK Antiangiogenesis strategies revisited: from starving tumors to alleviating hypoxia. Cancer Cell (2014) 26:605–22. 10.1016/j.ccell.2014.10.006 PMC4269830 25517747

[5] Osawa T Tsuchida R Muramatsu M Shimamura T Wang F Suehiro J Inhibition of histone demethylase JMJD1A improves anti-angiogenic therapy and reduces tumor-associated macrophages. Cancer Res (2013) 73:3019–28. 10.1158/0008-5472.CAN-12-3231 23492365

[6] Chang CH Qiu J O’Sullivan D Buck MD Noguchi T Curtis JD Metabolic Competition in the Tumor Microenvironment Is a Driver of Cancer Progression. Cell (2015) 162:1229–41. 10.1016/j.cell.2015.08.016 PMC4864363 26321679

[7] Pan M Reid MA Lowman XH Kulkarni RP Tran TQ Liu X Regional glutamine deficiency in tumours promotes dedifferentiation through inhibition of histone demethylation. Nat Cell Biol (2016) 18:1090–101. 10.1038/ncb3410 PMC5536113 27617932

[8] Tajan M Hock AK Blagih J Robertson NA Labuschagne CF Kruiswijk F A Role for p53 in the Adaptation to Glutamine Starvation through the Expression of SLC1A3. Cell Metab (2018) 28:721–36.e6. 10.1016/j.cmet.2018.07.005 30122553 PMC6224545

[9] Byun JK Choi YK Kim JH Jeong JY Jeon HJ Kim MK A Positive Feedback Loop between Sestrin2 and mTORC2 Is Required for the Survival of Glutamine-Depleted Lung Cancer Cells. Cell Rep (2017) 20:586–99. 10.1016/j.celrep.2017.06.066 28723563

[10] Huang D Li T Wang L Zhang L Yan R Li K Hepatocellular carcinoma redirects to ketolysis for progression under nutrition deprivation stress. Cell Res (2016) 26:1112–30. 10.1038/cr.2016.109 PMC5113304 27644987

[11] Hay N Reprogramming glucose metabolism in cancer: can it be exploited for cancer therapy? Nat Rev Cancer (2016) 16:635–49. 10.1038/nrc.2016.77 PMC5516800 27634447

[12] Altman BJ Stine ZE Dang CV From Krebs to clinic: glutamine metabolism to cancer therapy. Nat Rev Cancer (2016) 16:619–34. 10.1038/nrc.2016.71 PMC5484415 27492215

[13] Yang L Venneti S Nagrath D Glutaminolysis: A Hallmark of Cancer Metabolism. Annu Rev BioMed Eng (2017) 19:163–94. 10.1146/annurev-bioeng-071516-044546 28301735

[14] Daye D Wellen KE Metabolic reprogramming in cancer: unraveling the role of glutamine in tumorigenesis. Semin Cell Dev Biol (2012) 23:362–9. 10.1016/j.semcdb.2012.02.002 22349059

[15] Lane AN Fan TW Regulation of mammalian nucleotide metabolism and biosynthesis. Nucleic Acids Res (2015) 43:2466–85. 10.1093/nar/gkv047 PMC4344498 25628363

[16] Metallo CM Gameiro PA Bell EL Mattaini KR Yang J Hiller K Reductive glutamine metabolism by IDH1 mediates lipogenesis under hypoxia. Nature (2011) 481:380–4. 10.1038/nature10602 PMC3710581 22101433

[17] Akella NM Ciraku L Reginato MJ Fueling the fire: emerging role of the hexosamine biosynthetic pathway in cancer. BMC Biol (2019) 17:52. 10.1186/s12915-019-0671-3 31272438 PMC6610925

[18] Son J Lyssiotis CA Ying H Wang X Hua S Ligorio M Glutamine supports pancreatic cancer growth through a KRAS-regulated metabolic pathway. Nature (2013) 496:101–5. 10.1038/nature12040 PMC3656466 23535601

[19] Wise DR DeBerardinis RJ Mancuso A Sayed N Zhang XY Pfeiffer HK Myc regulates a transcriptional program that stimulates mitochondrial glutaminolysis and leads to glutamine addiction. Proc Natl Acad Sci U S A (2008) 105:18782–7. 10.1073/pnas.0810199105 PMC2596212 19033189

[20] Lowman XH Hanse EA Yang Y Ishak Gabra MB Tran TQ Li H p53 Promotes Cancer Cell Adaptation to Glutamine Deprivation by Upregulating Slc7a3 to Increase Arginine Uptake. Cell Rep (2019) 26:3051–60 e4. 10.1016/j.celrep.2019.02.037 30865893 PMC6510239

[21] Kamphorst JJ Nofal M Commisso C Hackett SR Lu W Grabocka E Human pancreatic cancer tumors are nutrient poor and tumor cells actively scavenge extracellular protein. Cancer Res (2015) 75:544–53. 10.1158/0008-5472.CAN-14-2211 PMC4316379 25644265

[22] Fu Q Xu L Wang Y Jiang Q Liu Z Zhang J Tumor-associated Macrophage-derived Interleukin-23 Interlinks Kidney Cancer Glutamine Addiction with Immune Evasion. Eur Urol (2019) 75:752–63. 10.1016/j.eururo.2018.09.030 30293904

[23] Gabrilovich DI Nagaraj S Myeloid-derived suppressor cells as regulators of the immune system. Nat Rev Immunol (2009) 9:162–74. 10.1038/nri2506 PMC2828349 19197294

[24] Si Y Merz SF Jansen P Wang B Bruderek K Altenhoff P Multidimensional imaging provides evidence for down-regulation of T cell effector function by MDSC in human cancer tissue. Sci Immunol (2019) 4:eaaw9159. 10.1126/sciimmunol.aaw9159 31628161

[25] Wang Y Yin K Tian J Xia X Ma J Tang X Granulocytic myeloid-derived suppressor cells promote the stemness of colorectal cancer cells through exosomal S100A9. Adv Sci (Weinh) (2019) 6:1901278. 10.1002/advs.201901278 31559140 PMC6755519

[26] Liang H Deng L Hou Y Meng X Huang X Rao E Host STING-dependent MDSC mobilization drives extrinsic radiation resistance. Nat Commun (2017) 8:1736. 10.1038/s41467-017-01566-5 29170400 PMC5701019

[27] De Henau O Rausch M Winkler D Campesato LF Liu C Cymerman DH Overcoming resistance to checkpoint blockade therapy by targeting PI3Kgamma in myeloid cells. Nature (2016) 539:443–7. 10.1038/nature20554 PMC5634331 27828943

[28] Wu WC Sun HW Chen HT Liang J Yu XJ Wu C Circulating hematopoietic stem and progenitor cells are myeloid-biased in cancer patients. Proc Natl Acad Sci U S A (2014) 111:4221–6. 10.1073/pnas.1320753111 PMC3964061 24591638

[29] Waight JD Netherby C Hensen ML Miller A Hu Q Liu S Myeloid-derived suppressor cell development is regulated by a STAT/IRF-8 axis. J Clin Invest (2013) 123:4464–78. 10.1172/JCI68189 PMC3784535 24091328

[30] Dolcetti L Peranzoni E Ugel S Marigo I Fernandez Gomez A Mesa C Hierarchy of immunosuppressive strength among myeloid-derived suppressor cell subsets is determined by GM-CSF. Eur J Immunol (2010) 40:22–35. 10.1002/eji.200939903 19941314

[31] Welte T Kim IS Tian L Gao X Wang H Li J Oncogenic mTOR signalling recruits myeloid-derived suppressor cells to promote tumour initiation. Nat Cell Biol (2016) 18:632–44. 10.1038/ncb3355 PMC4884142 27183469

[32] Pylayeva-Gupta Y Lee KE Hajdu CH Miller G Bar-Sagi D Oncogenic Kras-induced GM-CSF production promotes the development of pancreatic neoplasia. Cancer Cell (2012) 21:836–47. 10.1016/j.ccr.2012.04.024 PMC3721510 22698407

[33] Kumar V Patel S Tcyganov E Gabrilovich DI The Nature of Myeloid-Derived Suppressor Cells in the Tumor Microenvironment. Trends Immunol (2016) 37:208–20. 10.1016/j.it.2016.01.004 PMC4775398 26858199

[34] Wu WC Sun HW Chen J OuYang HY Yu XJ Chen HT Immunosuppressive immature myeloid cell generation is controlled by glutamine metabolism in human cancer. Cancer Immunol Res (2019) 7:1605–18. 10.1158/2326-6066.CIR-18-0902 31387898

[35] Wu C Ning H Liu M Lin J Luo S Zhu W Spleen mediates a distinct hematopoietic progenitor response supporting tumor-promoting myelopoiesis. J Clin Invest (2018) 128:3425–38. 10.1172/JCI97973 PMC6063469 29771686

[36] Schultze JL Mass E Schlitzer A Emerging Principles in Myelopoiesis at Homeostasis and during Infection and Inflammation. Immunity (2019) 50:288–301. 10.1016/j.immuni.2019.01.019 30784577

[37] Pinho S Frenette PS Haematopoietic stem cell activity and interactions with the niche. Nat Rev Mol Cell Biol (2019) 20:303–20. 10.1038/s41580-019-0103-9 PMC6483843 30745579

[38] Kuang DM Xiao X Zhao Q Chen MM Li XF Liu RX B7-H1-expressing antigen-presenting cells mediate polarization of protumorigenic Th22 subsets. J Clin Invest (2014) 124:4657–67. 10.1172/JCI74381 PMC4191045 25244097

[39] Sun HW Yu XJ Wu WC Chen J Shi M Zheng L GLUT1 and ASCT2 as Predictors for Prognosis of Hepatocellular Carcinoma. PloS One (2016) 11:e0168907. 10.1371/journal.pone.0168907 28036362 PMC5201247

[40] Mendez-Ferrer S Michurina TV Ferraro F Mazloom AR Macarthur BD Lira SA Mesenchymal and haematopoietic stem cells form a unique bone marrow niche. Nature (2010) 466:829–34. 10.1038/nature09262 PMC3146551 20703299

[41] Katayama Y Hidalgo A Furie BC Vestweber D Furie B Frenette PS PSGL-1 participates in E-selectin-mediated progenitor homing to bone marrow: evidence for cooperation between E-selectin ligands and alpha4 integrin. Blood (2003) 102:2060–7. 10.1182/blood-2003-04-1212 12763924

[42] Xiang Y Stine ZE Xia J Lu Y O’Connor RS Altman BJ Targeted inhibition of tumor-specific glutaminase diminishes cell-autonomous tumorigenesis. J Clin Invest (2015) 125:2293–306. 10.1172/JCI75836 PMC4497742 25915584

[43] Moreadith RW Lehninger AL The pathways of glutamate and glutamine oxidation by tumor cell mitochondria. Role of mitochondrial NAD(P)+-dependent malic enzyme. J Biol Chem (1984) 259:6215–21. 10.1016/S0021-9258(20)82128-0 6144677

[44] Han D Lerner AG Vande Walle L Upton JP Xu WH Hagen A IRE1 alpha Kinase Activation Modes Control Alternate Endoribonuclease Outputs to Determine Divergent Cell Fates. Cell (2009) 138:562–75. 10.1016/j.cell.2009.07.017 PMC2762408 19665977

[45] Urano F Wang XZ Bertolotti A Zhang YH Chung P Harding HP Coupling of stress in the ER to activation of JNK protein kinases by transmembrane protein kinase IRE1. Science (2000) 287:664–6. 10.1126/science.287.5453.664 10650002

[46] Wang L Perera BG Hari SB Bhhatarai B Backes BJ Seeliger MA Divergent allosteric control of the IRE1alpha endoribonuclease using kinase inhibitors. Nat Chem Biol (2012) 8:982–9. 10.1038/nchembio.1094 PMC3508346 23086298

[47] Cross BC Bond PJ Sadowski PG Jha BK Zak J Goodman JM The molecular basis for selective inhibition of unconventional mRNA splicing by an IRE1-binding small molecule. Proc Natl Acad Sci U S A (2012) 109:E869–78. 10.1073/pnas.1115623109 PMC3326519 22315414

[48] Fornes O Castro-Mondragon JA Khan A van der Lee R Zhang X Richmond PA JASPAR 2020: update of the open-access database of transcription factor binding profiles. Nucleic Acids Res (2020) 48:D87–92. 10.1093/nar/gkz1001 PMC7145627 31701148

[49] Bennett BL Sasaki DT Murray BW O’Leary EC Sakata ST Xu W SP600125, an anthrapyrazolone inhibitor of Jun N-terminal kinase. Proc Natl Acad Sci U S A (2001) 98:13681–6. 10.1073/pnas.251194298 PMC61101 11717429

[50] Gabrilovich DI Myeloid-Derived Suppressor Cells. Cancer Immunol Res (2017) 5:3–8. 10.1158/2326-6066.CIR-16-0297 28052991 PMC5426480

[51] Chiu DK Xu IM Lai RK Tse AP Wei LL Koh HY Hypoxia induces myeloid-derived suppressor cell recruitment to hepatocellular carcinoma through chemokine (C-C motif) ligand 26. Hepatology (2016) 64:797–813. 10.1002/hep.28655 27228567

[52] Yuen VW Wong CC Hypoxia-inducible factors and innate immunity in liver cancer. J Clin Invest (2020) 130:5052–62. 10.1172/JCI137553 PMC7524494 32750043

[53] Bobrovnikova-Marjon EV Marjon PL Barbash O Vander Jagt DL Abcouwer SF Expression of angiogenic factors vascular endothelial growth factor and interleukin-8/CXCL8 is highly responsive to ambient glutamine availability: role of nuclear factor-kappaB and activating protein-1. Cancer Res (2004) 64:4858–69. 10.1158/0008-5472.CAN-04-0682 15256456

[54] Zhang Y Lv D Kim HJ Kurt RA Bu W Li Y A novel role of hematopoietic CCL5 in promoting triple-negative mammary tumor progression by regulating generation of myeloid-derived suppressor cells. Cell Res (2013) 23:394–408. 10.1038/cr.2012.178 23266888 PMC3587709

[55] Oburoglu L Tardito S Fritz V de Barros SC Merida P Craveiro M Glucose and Glutamine Metabolism Regulate Human Hematopoietic Stem Cell Lineage Specification. Cell Stem Cell (2014) 15:169–84. 10.1016/j.stem.2014.06.002 24953180

[56] Panopoulos AD Watowich SS Granulocyte colony-stimulating factor: molecular mechanisms of action during steady state and ‘emergency’ hematopoiesis. Cytokine (2008) 42:277–88. 10.1016/j.cyto.2008.03.002 PMC2852428 18400509

[57] Alshetaiwi H Pervolarakis N McIntyre LL Ma D Nguyen Q Rath JA Defining the emergence of myeloid-derived suppressor cells in breast cancer using single-cell transcriptomics. Sci Immunol (2020) 5:eaay6017. 10.1126/sciimmunol.aay6017 32086381 PMC7219211

[58] Zhang B Wang Z Wu L Zhang M Li W Ding J Circulating and tumor-infiltrating myeloid-derived suppressor cells in patients with colorectal carcinoma. PloS One (2013) 8:e57114. 10.1371/journal.pone.0057114 23437326 PMC3577767

[59] Angell TE Lechner MG Smith AM Martin SE Groshen SG Maceri DR Circulating Myeloid-Derived Suppressor Cells Predict Differentiated Thyroid Cancer Diagnosis and Extent. Thyroid (2016) 26:381–9. 10.1089/thy.2015.0289 PMC4790214 26756227

[60] Shojaei F Wu X Qu X Kowanetz M Yu L Tan M G-CSF-initiated myeloid cell mobilization and angiogenesis mediate tumor refractoriness to anti-VEGF therapy in mouse models. Proc Natl Acad Sci U S A (2009) 106:6742–7. 10.1073/pnas.0902280106 PMC2665197 19346489

