# Supplementary material for: Glutamine Deprivation Promotes the Generation and Mobilization of MDSCs by Enhancing Expression of G-CSF and GM-CSF
Source: Front Immunol. 2021 Feb 2;11:616367. doi: 10.3389/fimmu.2020.616367 (PMC7884351; doi:10.3389/fimmu.2020.616367)
Supplement: Supplementary file 1 [file Table_1.docx]

Supplementary Material

for

Glutamine deprivation promotes the generation and mobilization of MDSCs by enhancing expression of G-CSF and GM-CSF

Hong-Wei Sun^1,2†^, Wen-Chao Wu^1†^, Hai-Tian Chen^3^, Yituo Xu^1,2^, Yanyan Yang^1,2^, Jing Chen^1^, Xing-Juan Yu^1^, Zilian Wang^3^, Ze-Yu Shuang^1,4*^, Limin Zheng^1,2*^

†These authors contributed equally to this work

* Corresponding author

**Supplementary Table S1** Summary of materials.

| **Materials** | **Manufacturers** | **Cat#** |
| --- | --- | --- |
| Lineage-FITC | BioLegend | 133302 |
| Sca-1-AF700 | eBioscience | 56–5981 |
| CD117-BV421 | BioLegend | 105827 |
| CD16/CD32-PE | BD Pharmingen | 553145 |
| CD34-AF647 | BD Pharmingen | 560230 |
| CD45-FITC | BD Biosciences | 553079 |
| CD29-PE-Cy7 | BioLegend | 102221 |
| Ly6G-PE-CF594 | BD Biosciences | 562700 |
| CD11b-PE-Cy7 | BD Biosciences | 561098 |
| Ly6C-AF647 | BioLegend | 128009 |
| CD8a-AF700 | BD Biosciences | 557959 |
| Ki67-PE | BD Pharmingen | 556027 |
| LEAF™ Purified anti-mouse CD3ε | BioLegend | 100314 |
| LEAF™ Purified anti-mouse CD28 | BioLegend | 102112 |
| anti-p-JNK Ab | CST | 4668T |
| anti-JNK Ab | CST | 9252T |
| anti-XBP1s Ab | CST | 12782S |
| anti-CHOP Ab | Abcam | ab179823 |
| anti-β-actin Ab | CST | 4967 |
| anti-CXCL12 Ab | Abcam | ab25117 |
| anti-G-CSF Ab | Santa Cruz | sc-49679 |
| RPMI | Thermo Fisher | C11875500BT |
| RPMI without glutamine | Thermo Fisher | 21870076 |
| FBS | Thermo Fisher | 10099-141 |
| Glutamine 100× | Thermo Fisher | 25030081 |
| Embryomax nucleosides mix | Merck-Millipore | ES-008-D |
| D-(+)-Glucosamine hydrochloride | Sigma-Aldrich | G1514-100G |
| MethoCult™ GF M3434 | Stemcell Technologies | 03434 |
| BPTES | ApexBio Technology | B6008 |
| DON | Sigma-Aldrich | D2141 |
| AOA | Sigma-Aldrich | C13408 |
| Anisomycin | Selleck | S7409 |
| SP600125 | Selleck | S1460 |
| APY29 | ApexBio Technology | B3288 |
| 4μ8C | Selleck | S7272 |
| TRI REAGENT | Invitrogen | AM9738 |
| 5X All-In-One MasterMix | ABM | G492 |
| THUNDERBIRD SYBR qPCR Mix | TOYOBO | QPS-201 |
| Immobilon Western Chemiluminescent HRP Substrate | Millipore | WBKLS0500 |

**Supplementary Table S2** Primer sequences.

| **Genes** | **Forward** | **Reverse** |
| --- | --- | --- |
| ACTB | CAGCCTTCCTTCTTGGGTATG | TGGCATAGAGGTCTTTACGGAT |
| G-CSF | GTCAGGACGAGAGGCCGTT | GGGGTGACACAGCTTGTAGGT |
| GM-CSF | CAGACATACTGCCCCCCAAC | TTACGCAGGCACAAAAGCAG |
| CXCL12 | CAGTGACGGTAAACCAGTCAGC | AGGGCACAGTTTGGAGTGTTG |
| KitL | GCGGGAATCCTGTGACTGAT | CATCCCGGCGACATAGTTGA |
| VCAM1 | CTGGGAAGCTGGAACGAAGT | GCCAAACACTTGACCGTGAC |
| ANGPT1 | CATCCAGGAGTTGGAGAAGCA | TCTCTTTTTCCTCCCTTTAGCA |
| SPP1 | TTCACTCCAATCGTCCCTACAG | CCTTAGACTCACCGCTCTTCAT |
